# Direct Observation of Secondary Nucleation in Huntingtin Amyloid Formation by High-Speed Atomic Force Microscopy

**DOI:** 10.1021/jacs.5c05571

**Published:** 2025-06-12

**Authors:** Chris van Ewijk, Greeshma Jain, Yari K. Knelissen, Sourav Maity, Patrick C. A. van der Wel, Wouter H. Roos

**Affiliations:** † Molecular Biophysics, Zernike Instituut, 3647 Rijksuniversiteit Groningen, 9747 AG Groningen, The Netherlands; ‡ Solid-State Nuclear Magnetic Resonance, Zernike Instituut, 3647 Rijksuniversiteit Groningen, 9747 AG Groningen, The Netherlands

## Abstract

Amyloid fibril formation is a hallmark of various neurodegenerative diseases such as Huntington’s (HD), Alzheimer’s, and Parkinson’s disease. The protein aggregation process involves slow nucleation events followed by rapid growth and elongation of formed fibrils. Understanding the pathways of amyloid formation is key to development of novel therapeutic agents that can interfere with the pathogenic protein misfolding events. Recent studies of aggregation by polypeptides from Alzheimer’s and Huntington’s disease have identified the importance of a poorly understood secondary nucleation process that may even be the dominant source of protein aggregate formation. Here, we focus on the polyglutamine-expansion disorder HD and employ mechanistic and structural studies to study different aspects of secondary nucleation in the aggregation of huntingtin Exon 1 (HttEx1). Notably, we apply high-speed atomic force microscopy (HS-AFM) to directly observe the process on the single-particle level and in real time. Our observations show unique features of the amyloid formation dynamics in real time, including secondary nucleation, elongation, and the formation of large bundles of fibrils as a result of nucleated branching. We examine the role of HttEx1 flanking segments during the aggregation process, revealing that the N-terminal Htt^NT^ segment exhibits a clear primary nucleation-aggregation-enhancing ability; however, it does not seem to induce or affect the secondary nucleation process. The obtained results illuminate the complex aggregation process of HttEx1 and have implications for attempts to inhibit or modulate it for therapeutic purposes.

## Graphical abstract

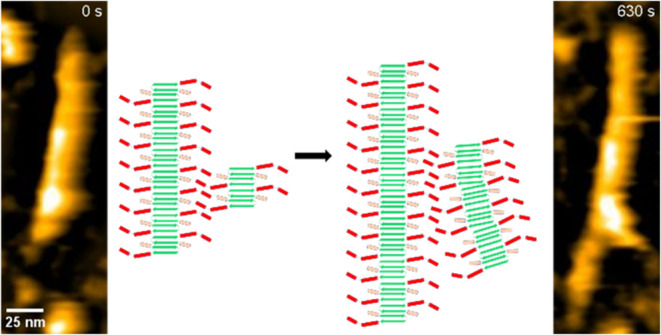

## Introduction

Aggregation of peptides and proteins into insoluble amyloid fibrils is a hallmark of various neurodegenerative diseases.^1^ Given the potential pathogenic role of these aggregates, there is a broad interest in understanding the molecular mechanisms of amyloid formation. Amyloid formation is typically considered to be a nucleation-based process that features two distinct phases: the initial phase where (primary) nucleation occurs, and the elongation phase where a rapid growth of existing fibrils can be observed.^2,3^ The rapid growth process traditionally was explained by elongation of existing fibril ends, combined with the progressive fragmentation of extending fibrils. Recently, quantitative analysis of fibril growth kinetics has shown that the produced fibrils serve as a nucleation point for new fibrils in a way that is not explained by fibril elongation alone.^4–9^ This process, called secondary nucleation, has been shown to even be a dominant aggregation mechanism for several different amyloid proteins implicated in disease.^4–6,10,11^ It is thought to reflect a process mediated by the fibril surfaces, and not just the fibril ends, but the details of its molecular origins remain under investigation. Besides its role in spontaneous aggregation, secondary nucleation is relevant to prion-like propagation, which is a cause of disease and may also be implicated in disease propagation. Consequently, there is an impetus toward preventing amyloid formation through the inhibition of secondary nucleation.^12–14^ However, for such an effort, a better understanding of the mechanistic details of secondary nucleation is vital, especially for the design of effective therapeutic agents.

Despite its important role, the secondary nucleation process remains poorly understood and is highly debated.^2,15^ To elucidate this process, we turn toward huntingtin (Htt) protein aggregates associated with Huntington’s disease (HD),^16^ since recent studies have identified secondary-nucleation-dominated aggregation.^7–9^ This neurological disease results from a heritable mutation that causes an expansion of a glutamine repeat region (polyQ) in the first exon of the huntingtin protein, leading to intraneuronal protein deposits.^17^ These deposits have been implicated in determining disease onset, progression and toxicity,^18–20^ although the precise impact is thought to depend on the nature of the inclusions.^18^ The cellular inclusions (in patients and model animals) contain N-terminal fragments of the Htt protein, which match the first exon (HttEx1).^17^ The generation of this HttEx1 fragment is driven by aberrant splicing or proteolytic events, which are both enhanced by the repeat expansion of the mutated gene.^21–23^ Consequently, HttEx1 generation increases progressively upon somatic expansion of the CAG repeat.^24^ HttEx1 features the (mutated) polyQ stretch, flanked by a 17-residue N-terminal segment (Htt^NT^) and a proline-rich C-terminal domain (PRD) (see Figure 1). These flanking segments impact on the amyloid formation process, and form a disordered fuzzy coat in the aggregates.^25–30^

**Figure 1:**
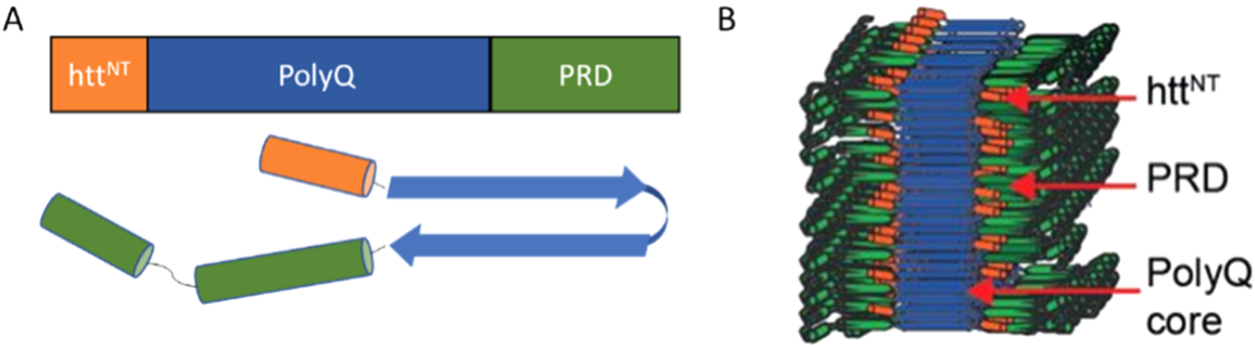
Huntingtin exon 1. (A) Primary and secondary structure of huntingtin exon 1 showing the N-terminal domain (Htt^NT^), the polyglutamine core (PolyQ) and the proline-rich domain at the C-terminal (PRD). (B) Model of HttEx1 amyloid fibril structure.

The exact role and effect of the flanking segments on the amyloid formation process remains however poorly understood. The Htt^NT^ segment is believed to play a vital role in primary nucleation, bringing the polyQ stretches in close proximity allowing for the misfolding into the β-sheet structure.^28,31^ However, there are multiple conceptions on the effect of the N-terminal domain on the aggregation propensity and morphology of HttEx1 fibrils. It has been reported to enhance HttEx1 aggregation,^7,29,32,33^ an effect which is even increased upon acetylation of Htt^NT^.^34^ Studies where Htt^NT^ was removed report varied effects, from a lack of bundled fibrils,^8,33,35^ to a relative increase of lateral association and clumping.^28^ It has also been suggested that parallel Htt^NT^ on the side of an existing fibril are the driving factors for secondary nucleation and subsequent branching.^31^ The reported polymorphism of HttEx1 fibrils is attributed to the fuzzy coat of the flanking domains and to local environmental conditions.^8,35–37^ Such polymorphism results in different levels of neurotoxicity, although a mechanistic explanation is still lacking.^37,38^ Speculations on the role of secondary nucleation on the emergence of polymorphism and toxicity of the deposits created high interest in understanding the underlying molecular processes to allow for possible control and inhibition of various aspects of amyloidogenic disorders which are currently incurable.

The aggregation behavior of HttEx1 shares common features with other amyloidogenic proteins, as β-sheet-rich nuclei are formed after a lag phase followed by a rapid increase of fibril lengths.^8,29,38^ The growth phase of HttEx1 involves a combination of elongation, fragmentation and secondary nucleation.^7–9^ By introducing preformed fibrils the slow lag phase can be bypassed, a “seeding” process which has been implicated to play a vital role in the disease progression of amyloid-related neurodegenerative disorders.^39,40^ Transmission of β-sheet-rich protein deposits between neurons may drive disease progression of HD patients.^39,41^ Unlike, for example, amyloid β 42, huntingtin amyloids form bundles of fibrils with a branched structure as observed by atomic force microscopy (AFM)^9,42^ and electron microscopy (EM).^8,36^ It has been speculated that the bundles of HttEx1 fibrils are not formed due to lateral association of existing filaments, but may be a result of secondary nucleation on a fibril surface,^9^ which implies that secondary nucleation plays a crucial role in the polymorphism of HttEx1 fibrils. Combined, these features make HttEx1 an appealing system to study the elusive secondary nucleation process in amyloid formation.

To elucidate the huntingtin amyloid formation process we turn toward high-speed atomic force microscopy (HS-AFM), a single particle technique that allows for label-free, real-time observation of dynamic molecular processes with subsecond temporal resolution.^43–45^ HS-AFM has been deployed before to study the formation of other amyloid fibrils, e.g., those implicated in Alzheimer’s disease.^46–49^ Here, we use HS-AFM to capture HttEx1 amyloid formation, observing elongation and secondary nucleation in amyloid formation in real time and exploring the role of Htt^NT^ in these processes.

## Materials and Methods

### Protein Expression and Purification

A fusion protein construct having maltose-binding protein (MBP) as a solubility tag was used to produce Q32- and Q44-HttEx1 monomers, as previously described.^8,25^ The MBP-Q32-HttEx1, having 32 consecutive glutamines in polyQ domain was subcloned into a pMALc5x plasmid, whereas MBP-Q44-HttEx1, with 44 consecutive glutamines in polyQ domain was subcloned into pMALc2x plasmid by Genscript (Piscataway, NJ). These constructs were expressed in Escherichia coli BL21­(DE3) pLysS cells (Invitrogen, Grand Island, NY) and the cells were grown in LB medium with ampicillin and chloramphenicol at 37 °C. The protein expression was induced by adding 0.6–0.8 mM of IPTG (Sigma) to the cell culture and shaking at 18 °C for 16 h. The cells were collected by pelleting them at 5250*g* for 20 min. The cell lysis was done by suspending the cells in cold Phosphate Buffer Saline (PBS), pH 7.4 buffer with 0.5 mg/mL of lysozyme, 0.01% sodium azide and 1 Pierce EDTA-free protease inhibitor tablet (ThermoFisher Scientific). This suspension was then sonicated to burst the cells using VCX130 Vibra-cell sonicator, Sonics & Materials, Inc. by applying 70% amplitude for 20 min (pulse alternation—10s pulse, 10s break). The cell debris was removed by centrifuging the lysate at 125,000*g* for 45 min. The supernatant containing soluble fusion protein was collected, filtered with 0.45 μm syringe filters and purified using a HisTrap HP nickel column (GE Healthcare) on an Akta FPLC system. The protein was eluted using an imidazole gradient (from 10 to 75%) and the protein presence and purity was verified by 12% SDS-gel as described in.^8^ The purified fused protein was collected and imidazole was removed using dialysis (Dialysis tubing, MWCO 12.4 kDa, Sigma-Aldrich). To determine the protein concentration, the absorbance was measured at 280 nm with a JASCO V-750 spectrophotometer. The absorbance was measured in triplicate and average of the absorbance values (*n* = 3) was taken and divided by the extinction coefficient of 66,350 M^–1^ cm^–1^ (derived from the fusion protein sequence by ProtParam tool by ExPASy^70^). The molecular weight was verified by ESI-TOF mass spectrometry.^8^

### Protein Aggregation

MBP-Q32-HttEx1 and MBP-Q44-HttEx1 were cleaved to release soluble HttEx1 by using Factor Xa (FXa; Promega, Madison, WI) at 400:1 (Fused protein: Protease) molar ratio. To generate ΔN15-Q44-HttEx1, the MBP-Q44-HttEx1 fusion protein was cleaved by Trypsin (*N*-tosyl-l-phenylalanine chloromethyl ketone treated trypsin lyophilized powder from bovine pancreas), redissolved in PBS buffer with 3 μM HCl at 50:1 (Fused protein/Protease) molar ratio. The protein cleavage was carried out at room temperature and was monitored by SDS-PAGE (Bio-Rad Mini-Protein Precast TGX Gels 12%). The final protein aggregates were collected after 4 days, and pelleted down by centrifuging at 2400*g* for 20 min. The supernatant was removed and the protein aggregates were washed three times with PBS buffer to ensure complete removal of MBP. The pre-existing Q44-HttEx1 fibril seeds used in AFM and kinetics measurements were prepared by aggregating 50 μM MBP-Q44-HttEx1 fused protein with FXa protease at 400:1 (Fused protein: Protease) molar ratio. The aggregation was performed at room temperature for 96 h. After that, the protein aggregates were washed three times using PBS buffer.

### High-Speed Atomic Force Microscopy

Atomic force microscopy was performed using an SS-NEX High-Speed Atomic Force Microscope (RIBM, Japan) in amplitude modulation tapping mode in liquid. Ultrashort cantilevers with high resonance frequency (USC-F1.2-k0.15, Nanoworld, Switzerland) were used. Short Q44-HttEx1 fibrils (seeds), which were preformed at room temperature, were incubated on mica surface for 10 min, and subsequently washed with PBS. The sample is then placed in the recording chamber containing the cantilever in a solution containing 60 μM MBP-Q44-HttEx1 cleaved with either FXa at 400:1 molar ratio (HttEx1/FXa), or trypsin 50:1 (HttEx1/Trypsin) molar ratio. Images were recorded every 5 s, unless stated otherwise. At *t* = 0 s AFM imaging started. All images were processed using ImageJ^71^ software with additional home-written plugins.

### Thioflavin T (ThT) Assay

The aggregation kinetics were measured using 20, 30, 50 and 60 μM MBP-Q32-HttEx1, with cleavage by FXa at 400:1 molar ratio (HttEx1/FXa). These ThT assays were performed with the ThT present during the aggregation reaction, using fluorescence plate readers, as described next. For kinetics assays, the 60 μM MBP-Q44-HttEx1 was divided in two batches: the first batch was treated with FXa at 400:1 molar ratio (HttEx1/FXa) to study the Q44-HttEx1 aggregation kinetics, and the other batch was treated with Trypsin (*N*-tosyl-l-phenylalanine chloromethyl ketone treated trypsin lyophilized powder from bovine pancreas, redissolved in PBS buffer with 3 μM HCl) at 50:1 molar ratio (HttEx1/trypsin) to study the ΔN15-Q44-HttEx1 aggregation kinetics. The effects of seeds on aggregation kinetics of 67.5 μM fused Q44-HttEx1 were measured by cleaving it by 400:1 molar ratio (HttEx1/FXa), adding seeds of preformed Q44-HttEx1 fibrils made at room temperature. The seed concentration used for the assay was 10%. A 1 mM ThT stock solution was made in DMSO and the final concentration of ThT in the well was 15 μM. The experiments were carried out in black polystyrene, clear bottom, 96-well plates (Corning) and measured on a TECAN Spark 10 M microplate reader. The excitation wavelength is 442 nm and the emission wavelength is 484 nm, with excitation and emission bandwidths of 10 nm. The experiments were done in triplicate.

### Negative-Stain TEM

Negative-stain transmission electron microscopy (TEM) was performed on samples from the Q32-HttEx1 aggregation process, at respective time intervals (2, 20, 71 and 96 h) (Figure S3A–C), as well as the final Q44-HttEx1 (Figure 2C), and seeded and unseeded Q44-HttEx1 fibrils (Figure S2C–D). The time dependent samples were frozen on specific time points in liquid nitrogen, and thawed at room temperature for making grids. The moment the sample was thawed, it was quickly mixed and placed on the grid. The plain carbon support film—200 mesh copper grids (SKU FCF200-Cu-50, Electron Microscopy Sciences, Hatfield, PA) were glow discharged for 0.5–1 min and then the fibril samples were deposited on the grid for 0.5–1 min. The excess sample was removed by blotting and then the negative staining agent 2% (w/v) uranyl acetate was allowed to stain the grid for 0.5–1 min. The excess stain was removed by blotting and the grid was air-dried. The grid was imaged on Philips CM120 electron microscope operating at 120 kV with a slow scan CCD camera (Gatan). Fiji,^72^ was used to measure the fibril widths, analogous to prior work.^8^ Each measurement was transverse to the fibril long-axis, and was the measure of the negative stained area of the fibril. In TEM images shown, the fibrils widths were determined in the regions where the fibril boundaries are clearly defined. The fibril widths were determined on isolated fibrils, using Plot profile tool in Fiji which plots the average grayscale intensity. We obtained three measurements per fibril, with exception to the fibrils where the width varied significantly. For the fibrils that varied in width, we did more than three measurements per fibril.

**Figure 2:**
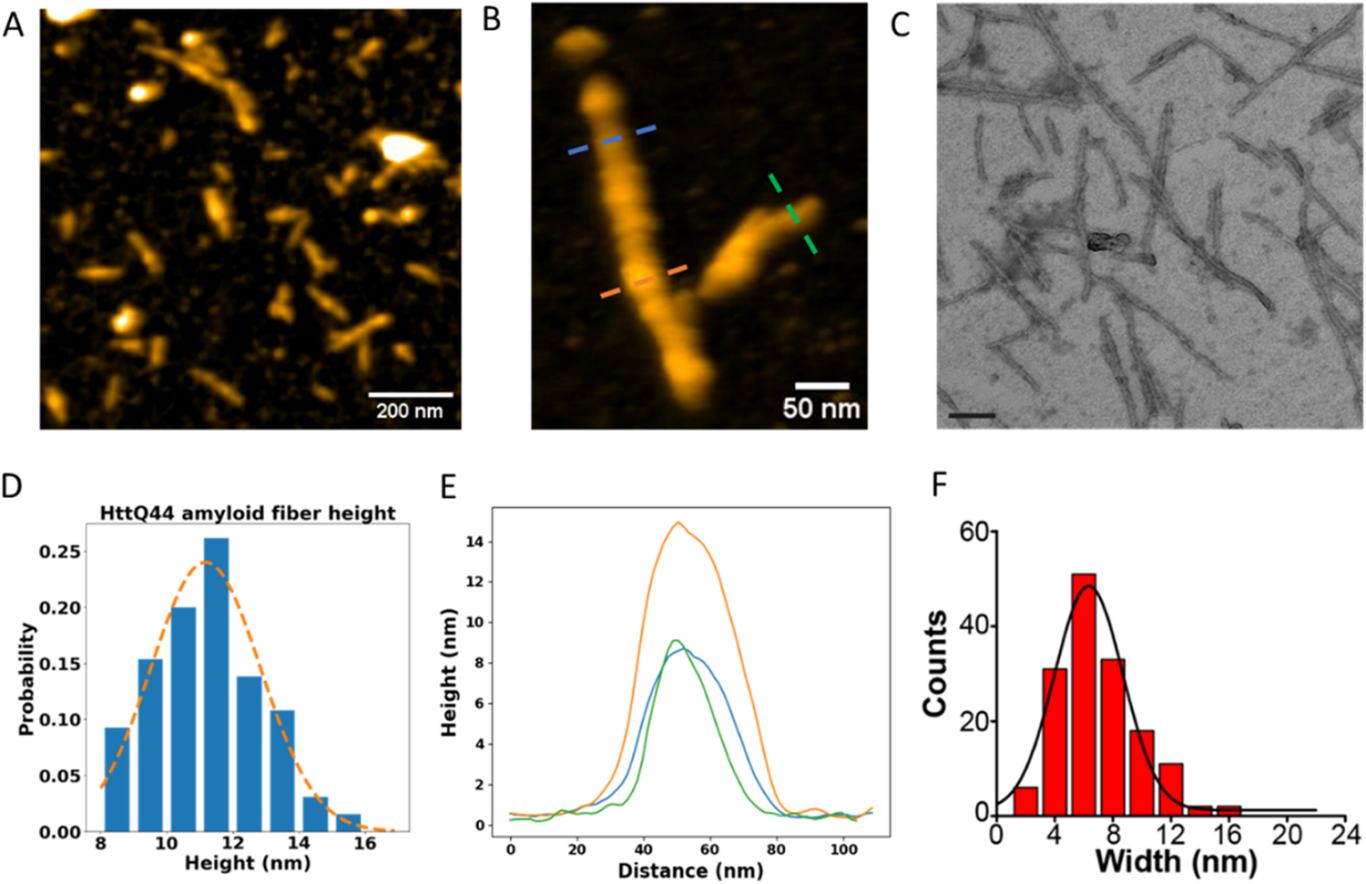
Q44-HttEx1 amyloid fibril morphology by HS-AFM and EM. (A) HS-AFM overview image of 60 μM Q44-HttEx1 fibrils on a mica surface. (B) Close up of Q44-HttEx1 amyloid fibrils. (C) Negative stain TEM image of 60 μM Q44-HttEx1 fibrils formed at room temperature. Scale bar 100 nm. (D) Distribution of Q44-HttEx1 amyloid fibril height by HS-AFM with an average of 11 ± 2 nm. (E) Height profiles taken along the drawn lines in (B). (F) Distribution of Q44-HttEx1 fibril widths as measured by TEM with an average of 6 ± 2 nm.

## Results

### Secondary Nucleation Plays a Key Role in HttEx1 Fibril Formation

To study the mechanistic aspects of HttEx1 aggregation in vitro, kinetic studies were performed. Amyloidogenic HttEx1 was produced in an aggregation-resistant state as a fusion protein, using maltose binding protein (MBP) as a solubility tag. In this setup, the aggregation can be triggered on demand by the addition of factor Xa (FXa) protease, which releases HttEx1 to allow it to aggregate into amyloid fibrils (see Figure 1A,B).^8,50^ In prior work, we established that Q44-HttEx1 aggregation follows a misfolding pathway dominated by secondary nucleation.^8^ A similar fluorometric thioflavin T (ThT) assay of varying concentrations (20 to 60 μM) of Q32-HttEx1 protein was used to analyze its molecular mechanism of aggregation (see Figure S1). A clear concentration dependence is visible, with variations in the lag phase time, half-times and slopes (Figure S1C). Plotting the half-time versus the log of the monomer concentration gives a linear trend (see Figure S1A), which indicates that the dominant aggregation mechanism does not change with monomer concentration. Therefore, one can apply a global fit approach,^51^ in which the different curves for different concentrations are fitted to a single aggregation model. Candidate models include the processes of primary nucleation, fibril elongation, and (optionally) secondary nucleation (Figure S1B–D). The elongation and secondary nucleation processes both depend on the concentration of formed fibrils, which increases over time during the (spontaneous) aggregation process. Models without secondary nucleation are unable to explain our data (see Figure S1C–D), showing that secondary nucleation is important for the aggregation of unseeded Q32-HttEx1.^51^ The parameters of the fitting (Table S1) show also that the rate parameter for secondary nucleation (*k*_2_) is larger than that for spontaneous nucleation (*k*_n_). Here the secondary nucleation process reflects the catalytic role of the total fibril mass, rather than merely the number of fibril ends. It is important to stress that in this analysis these fibrils are the aggregates formed in the de novo aggregation reaction. In the case of nucleation-limited aggregation mechanisms, the aggregation process should be accelerated by adding preformed fibril seeds. Indeed, a significant reduction in the lag phase and acceleration in fibril formation was observed upon the addition of preformed fibrils (Figure S2A). The seeded fibril growth is expected to be based on the same fibril elongation and fibril-induced secondary nucleation processes present in de novo aggregation. Thus, HttEx1 aggregation is a prime example of an amyloid formation process in which there is a central role for secondary nucleation, in line with prior studies.^7–9^

### Morphology of HttEx1 Aggregates by TEM and AFM

To have a better understanding of the structural transformations of HttEx1 during the aggregation process, monitored by ThT above, we analyzed different time points by negative stain transmission electron microscopy (TEM). Samples from an aggregation experiment with 51 μM Q32-HttEx1 were taken and frozen in liquid nitrogen. TEM analysis showed the gradual growth of typical HttEx1 fibrils over time (see Figure S3A). Small fibrillar and globular species were observed in the lag phase of the aggregation process (2 h). The globular species were heterogeneous with a varying diameter of 12–30 nm (see Figure S2D), similar to oligomers seen in previous studies.^52,53^ The fibril widths, seen as indicators of HttEx1 fibril polymorphism,^8,25^ were 6 ± 2 nm (*n* = 71) (see Figure S3A,B). By 20 h, we observed that the amount of visible fibrils increased, at the expense of the observed globular species, consistent with the ThT assay results (Figure S1A). The width of these fibrils was 11 ± 2 nm (*n* = 104). Over time, the appearance of the growing fibrils changed, with the width approaching 9 ± 3 nm (*n* = 222) at 71 h. At later stages, the fibril width did not vary, but their length increased with time. The mature Q32-HttEx1 fibrils after 96 h had fibril widths of 8 ± 2 nm (*n* = 166). The mature fibrils that formed at the end of the aggregation process appeared qualitatively similar for Q32- and Q44-HttEx1 (see Figures S2 and S3), except for differences in width expected for changes in the polyQ segment length. Note that the width observed in the negative stain TEM analysis is thought to represent the dimensions of the fibril core (formed by the polyQ segment alone), as the stain cannot penetrate it.^8,54^

Thus, TEM can provide good-resolution images of the fibril morphology, but it is less suitable for probing the surface characteristics of the fibrils. To allow more detailed visualization of the fibril surface, mature Q44-HttEx1 fibrils were characterized by AFM (see Figures 2A,B and S4). The average height of 11 ± 2 nm (*n* = 65) of Q44-HttEx1 fibrils as measured by HS-AFM (See Figure 2D,E) compares well to results from TEM analysis of fibril widths (6 ± 2 nm, *n* = 154; Figure 2C,F), considering the latter is thought not to include the flanking segments.^54^ Here we note that we focus on the fibril height for the AFM analysis, since the apparent width of fibrils in AFM images is convoluted due to the shape of the AFM tip, making width measurements much less precise than height measurements in AFM.

Various aggregate-like structures are visible along the length of the fibrils (see Figure 2B,E), indicating that the surface of the fibrils is prone to associate with free monomers or small oligomers. The height distribution in Figure 2D does not include these higher aggregates. It is worth noting that HttEx1 fibrils are well-known to be more heterogeneous and less smooth than other amyloid fibrils, e.g., shown by AFM, cryo-EM and cryo-electron tomography.^9,35,36^

### Real-Time Imaging of HttEx1 Fibril Growth by AFM

Static AFM images could not answer the question of how these characteristic fibril structures form during HttEx1 aggregation. To probe this, the seeded aggregation process of Q44-HttEx1 was followed over time using HS-AFM. We focused on seeded aggregation (i.e., in the presence of preformed aggregates) as an efficient way to study the elongation and secondary nucleation processes. The aggregation process was started upon addition of a solution containing freshly cleaved protein monomers to the surface adhered seeds. This allowed for observing the process of fibril elongation in real time. The length increase of the fibril seeds was captured by HS-AFM, as displayed in Figure 3A,B. The quantification of bidirectional elongation rates of the seeds, showed an average of 5 ± 3 nm min^–1^ (see Figure 3C), although occasionally one side of a fibril did not elongate at all. Fibril elongation was generally not continuous over the course of the measurements, with phases of rapid growth alternated by stagnation phases (see Figure 3B).

**Figure 3:**
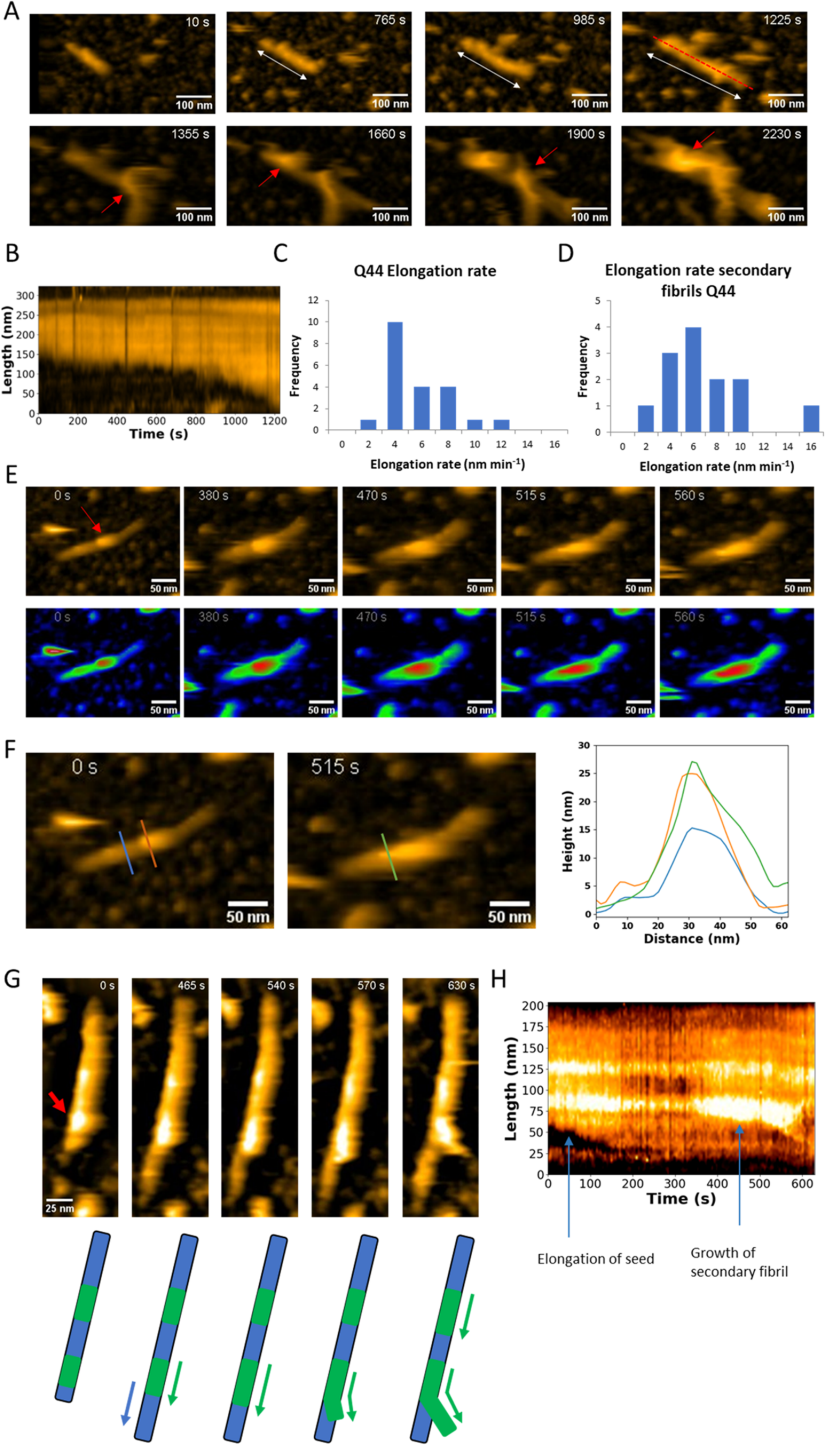
Seeded huntingtin amyloid formation in real time by HS-AFM displaying elongation and secondary nucleation. (A) Snapshots of Q44-HttEx1 amyloid formation showing elongation and nucleated branching. White double-sided arrows indicate the elongation of the fibril and red arrows indicate the appearance of secondary nucleated fibrils. Z-scale 0–1225s 35 nm, 1355–2230s 80 nm. (B) Kymograph along the dashed red line in (A) showing fibril elongation. (C) Elongation rates of Q44-HttEx1 fibrils. Average: 5 ± 3 nm min^–1^ (*n* = 21). (D) Elongation rates of secondary Q44-HttEx1 fibrils. Average: 6 ± 3 nm min^–1^. (*n* = 13). (E) Secondary nucleation event on the surface of an Q44-HttEx1 amyloid fibril. The red arrow points at the site of the secondary nucleation event. In the lower panels, the parent fibril and secondary fibril are colored in green and red respectively showing how the secondary fibril elongates. (F) Cross sections of the growing secondary fibril shown in panel (E). The blue line shows the height of the original fibril and the orange and green lines the height of the secondary nucleated fibril. (G) Snapshots of branching of secondary fibrils and corresponding schematic. The green segments indicate the secondary fibrils. (H) Kymograph along the fibril in (G) showing the growth of the fibril seed and secondary fibril.

### Observing HttEx1 Secondary Nucleation by AFM

Unlike bulk ThT assays, HS-AFM results allow the observation of molecular events underpinning the overall aggregation kinetics. One striking observation is the detection of what appears to be secondary nucleation events on the fibril surface, followed by the growth of new fibrils (see Figure 3A,E,F). Initially, small aggregates are observed on the fibril surface, manifesting as a sudden increase in the local fibril height. The red arrow in Figure 3E points at such an aggregate. Over time, these new features are seen to elongate, adopting a morphology that resembles a fibril structure, with the height of the secondary fibrils similar to that of singular fibrils (see Figure 3F). In Figure S5 a schematic of the growth of the secondary fibril is shown. Elongation of these newly formed fibrils occurs along the surface of the parent fibril (Figures S5 and S6). On approximately 90% of the fibrils such a secondary nucleation event is observed during the course of extended imaging (∼2000 s). Just like the observed seed fibril elongation (Figure 3A), growth of the daughter fibril formed by secondary nucleation also typically occurs bidirectionally. Also here, however, occasionally one side of a fibril did not elongate. The elongation of the secondary nucleated, newly formed fibril was tracked and is represented in Figure S7. The rate of elongation has an average value of 6 ± 3 nm min^–1^ (Figure 3D), which is similar to the rate of elongation of the original seed fibrils (Figure 3C). Eventually, the growing end of the new fibrils detach from the fibril surface, resulting in a branched structure (Figures 3G and S6). Secondary nucleation occurs both on the seeds as well as on newly grown parts of the seeded fibrils. Figure S8 shows an example of how a seed elongates on the surface and the appearance of a secondary nucleation event on the newly grown part. The secondary nucleated fibril grows and finally branches away. Multiple secondary nucleation events have been observed along a single fibril to finally appear as an unordered broom-like structure, with the fibrils sticking out at the end of the cluster (see Figure S9). As noted above, this type of morphology seems characteristic of HttEx1 fibrils. The fact that multiple fibrils originate from a single seed correlates well with the exponential growth observed in the ThT assays, and provides a molecular rationale for the secondary nucleation process identified by kinetic analysis. Secondary nucleation occurred on almost all of the fibril seeds over time, indicating the high susceptibility of the fibril surfaces to catalyze fibril nucleation events. In an attempt to quantify the amount of amyloid material, we measured the relative volumetric increase over time, which showed an increasing trend, as ThT assay curves also show (see Figure S10).

No primary nucleation events were observed during the time scales at which elongation and secondary nucleation were captured. These findings are consistent with the bulk aggregation mechanism (see Figure S1 and Table S1), which identified a dominance of secondary nucleation. It also fits with previous findings.^8,9,39^ Notably, there was no indication of larger preformed aggregates attaching to the fibril end during elongation or secondary nucleation, implying that elongation occurs by attachment of single monomers or small oligomers.

### On the Role of the Flanking Domains in Secondary Nucleation

The prevalence of secondary nucleation and branching shifted our attention to the nature of the interaction between free monomers and the fibril surface. An interesting aspect of HttEx1’s aggregation behavior is that it is not only dependent on the length of the polyQ domain, but also the flanking segments Htt^NT^ and PRD. Htt^NT^ is generally expected to play an important driving force in the aggregation process, dramatically shortening the lag phase of aggregation (at a given protein concentration).^7,26,28,29^ This implies a direct involvement in primary and/or secondary nucleation, consistent with a report that Htt^NT^-truncated HttEx1 constructs show a lack of lateral associations and branching.^33^ The role of Htt^NT^ can be studied by its removal prior to aggregation^8^ yielding the construct ΔN15-HttEx1 (see Figure 4A). The truncated HttEx1 aggregates slower than regular HttEx1 (see Figure 4B), consistent with prior studies,^8,28,29,55,56^ resulting in a curve that is more similar to that of Q32-HttEx1 than Q44-HttEx1. Next, HS-AFM was used to analyze the fibril growth by the truncated ΔN15-Q44-HttEx1 by HS-AFM (see Figure 4C,D). Interestingly, secondary nucleation, often followed by branching, was also observed in these samples. SI Movie S1 and Figure S11 shows an example of growth of a fibril from a seed and a secondary nucleation event that occurs on top of the newly grown part of the seed. On approximately 88% of the fibril seeds such a secondary nucleation event is observed, which is similar to the percentage observed on the Q44-HttEx1 fibrils. Bidirectional elongation of the fibrillar structures was observed with an average rate of 5 ± 1 nm min^–1^ (*n* = 9), whereas secondary nucleated ΔN15-Q44-HttEx1 fibrils elongated with an average rate of 6 ± 1 nm min^–1^ (*n* = 6) corresponding well to the growth rates of the normal Q44-HttEx1 fibrils. A specific example of nucleated branching with an accompanying schematic is depicted in Figure 4D (see also SI Movie S2). Initially, small aggregates are visible on the fibril surface (green segments in Figure 4D). These aggregates slowly grow in size to eventually take up a fibrillar structure. After elongating along the fibril, the growing end detaches from the parent-fibril, resulting in a branched structure. The process of nucleated branching occurs with a similar frequency as in Q44-HttEx1 experiments. Lateral association of fibrils and growth on the surface of existing fibrils is therefore not solely the result of Htt^NT^ mediated association.

**Figure 4:**
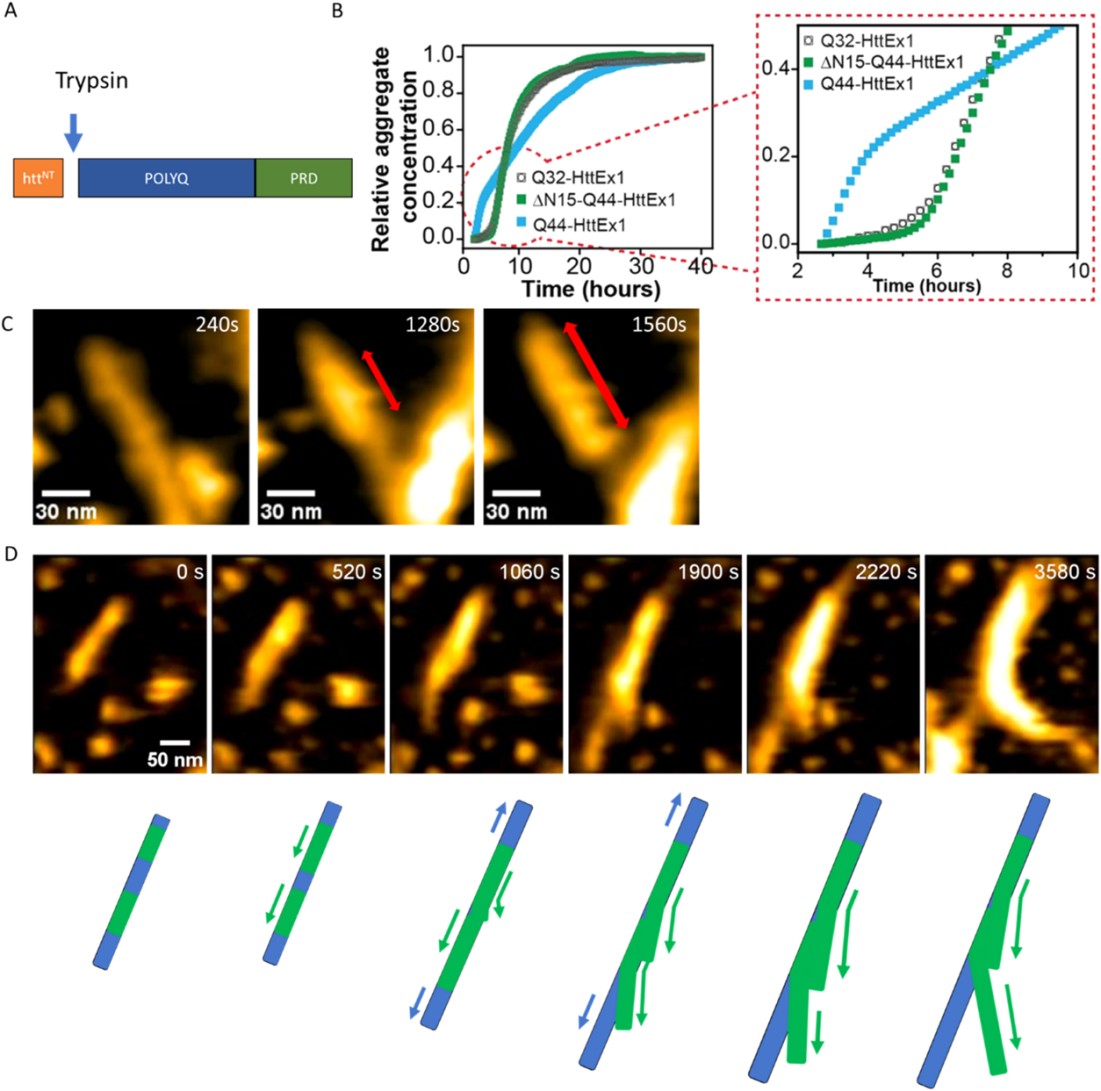
Real-time dynamics of seeded ΔN15-Q44-HttEx1 amyloid formation. (A) Schematic ΔN15 Structure showing the cleavage site of Trypsin. (B) Comparative aggregation kinetics of Q32-, Q44- and ΔN15-Q44-HttEx1, at 60 μM concentration, along with enlarged view of the initial lag and growth phase. All ThT data are measured in triplicates at 29 °C and the mean of the triplicates is plotted. (C) Snapshots of HS-AFM experiments showing secondary nucleation on a Δ15-Q44-HttEx1 amyloid fibril. (D) Snapshots with corresponding schematic of secondary nucleation and subsequent branching on a Δ15-Q44-HttEx1 amyloid fibril. The blue fibril represents the parent fibrils and the green fibrils are secondary nucleated fibrils. The snapshots are taken from SI Movie S2.

## Discussion

The notable role of secondary nucleation in the aggregation kinetics of HttEx1 was confirmed and visualized at the single fibril level in real time through the use of HS-AFM. These results are in line with prior bulk and static studies on HttEx1 aggregation.^8,9^ We observed the complementary effects of polyQ length and flanking domains, in the complex aggregation pathway of these proteins.^29,56^ Morphological analysis by EM at various time intervals unveiled the gradual growth of amyloid-like fibrils, following initial formation of prefibrillar globular species. The globular species were attributed to prefibrillar oligomers described in earlier studies.^52,53^ Analysis of the fibril concentration via ThT-assay enabled the dissection of the fibril formation mechanism, which pointed to the prominence of secondary nucleation in this process. Next we probed the molecular underpinnings of the huntingtin amyloid formation in real time by HS-AFM, revealing the elongation and secondary nucleation pathways on the single-particle level. The elongation of primary fibrils showed phases of rapid growth alternated by stagnation phases. A similar behavior was recently reported for Aβ42 fibrils,^49^ indicating that this might be inherent to amyloid formation. Consistent with bulk kinetics analyses, secondary nucleation of HttEx1 fibril formation was observed to occur on the sides of existing amyloid fibrils, being more common than direct primary nucleation. Thus, once fibrils are present (past the primary nucleation phase), this surface-mediated process appears to be a main driver of new fibril formation. Notably, this provides a direct observation of a molecular mechanism behind the concept of secondary nucleation: that the kinetics of fibril growth show a dependence on the total fibril mass, not just the number of growing ends. In our conventional understanding of (seeded) amyloid growth, the fibril ends would be considered the main (or only) source of fibril growth (aside from spontaneous primary nucleation events). The HS-AFM results highlight the importance of the available fibril surface area. The more fibrils that are already present, the more fibril surface area is available to capture the free available monomers, which will then misfold into the amyloid structure.

Due to these processes, small and isolated Q44-HttEx1 seeds were observed to grow into large bundles of fibrils as a result of monomer addition and subsequent branching. The appearance of large bundles due to secondary nucleation corresponds well to predictions from previous studies,^9^ where large bundles of fibrils were speculated to be a result of secondary nucleation rather than lateral association of existing fibrils. However, the driving force of branching remains uncertain. Nucleated branching has been speculated to result from a buildup of strain in the newly growing segment or steric constraints along the fibrils surface. Notably, the fibrils never completely separated in the analyzed HS-AFM data. Thus, the different filaments remain attached together, forming higher-order structures reminiscent of fibril clusters seen in vitro^36^ as well as in cells.^57^ Interestingly, this could imply that a single primary nucleation event could be behind the formation of entire μm-sized intracellular pathological inclusion bodies.^58^ Figure 5 provides a schematic visualization of such processes happening on the surfaces of fibrils formed from (first) primary nucleation, and (later) secondary nucleation.^8^

**Figure 5:**
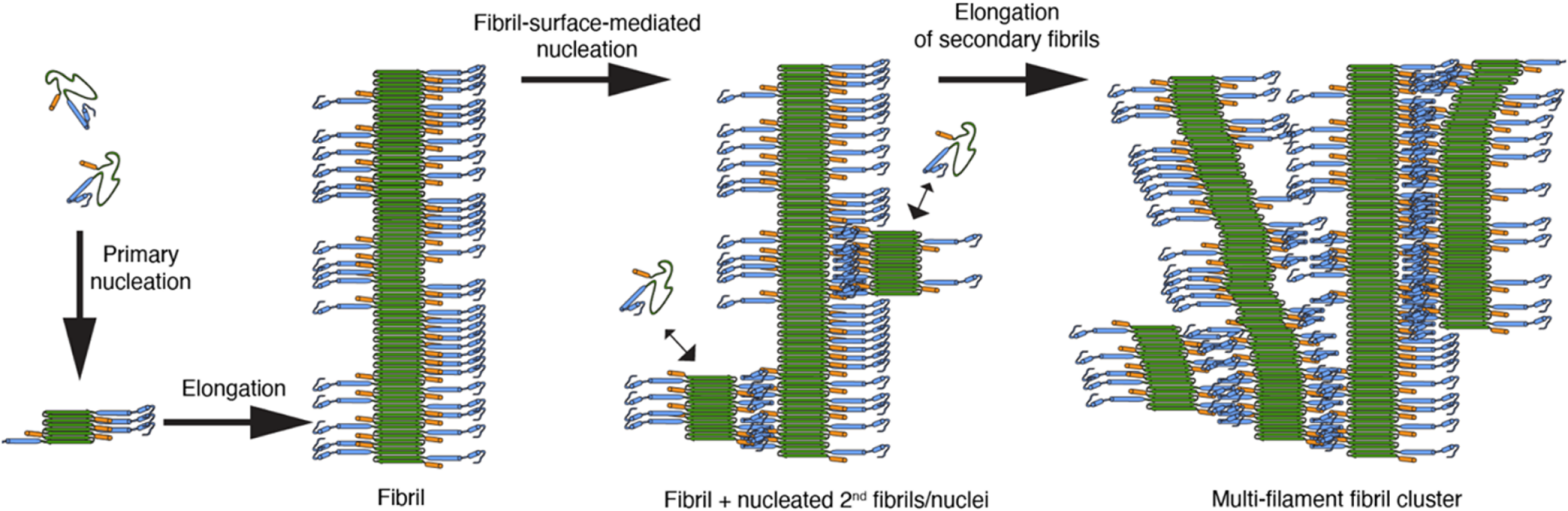
Schematic model for fibril assembly and secondary nucleation. Primary nucleation leads to the growth of an initial fibril (left). The fibril surface then serves as a catalytic site for the formation of secondary fibrils (secondary nucleation), via flanking domains (shown), the polyQ surface (not shown), or both. As a result of multiple secondary nucleation events on the fibril surfaces a multifilament fibril cluster can form. Color coding: green: polyQ β-strand core; blue: PRD; orange: Htt^NT^ segment. The model is an extended version of our model as previously published in ref 8.

Our results show that secondary nucleation happens along the fibril surface with no apparent specific sites, since multiple nucleation events can be observed on a single fibril, which all subsequently grow. The observed nucleation sites did not diffuse along the fibril, which may indicate a reasonably strong adhesion between the seed fibril and the newly formed assembly. It is worth noting that we cannot exclude the possibility of monomers diffusing over the fibril surface,^59^ and nucleation resulting when the local concentration increases. The nucleation sites appeared gradually on the fibril surface, indicating that for Q44-HttEx1 the nucleation does not occur by association of large oligomers formed in solution to the fibrils. Instead, the nucleation of monomers into oligomers and finally the structural conversion to amyloidogenic form all seem to happen along the fibril surface for secondary nuclei.

Both fibril elongation as well as secondary nucleation and branching are still prevalent for HttEx1 deprived of Htt^NT^. One implication seems to be that the known aggregation-enhancing role of this flanking segment is not (primarily) derived from its role in secondary nucleation. This fits with prior models,^7,26,29^ which attribute the effect of Htt^NT^ to derive primarily from its effect on early stages of spontaneous aggregation (i.e., related to primary nucleation rather than secondary nucleation). Nonetheless, Htt^NT^ might still play a role in the surface reactivity of HttEx1 fibrils, yet it is not the solely responsible trigger for secondary nucleation. Our findings are in contrast with the results from Shen et al.,^33^ who reported that removing Htt^NT^ results in a lack of fibril bundling. On the contrary, Vieweg et al.^28^ subsequently reported that removing Htt^NT^ induced a significant increase in lateral association and clumping of HttEx1 fibrils. Differences between previous studies may result from the different constructs used in preparation of the fibrils. Polymorphism of HttEx1 fibrils is known to be sensitive to the preparation conditions, differences in flanking segments and attached tags.^60^

An important question relates to the part(s) of the amyloid structure that are responsible for the secondary nucleation on the surface. Here, it is important to revisit the architecture of HttEx1 fibrils, which feature a solid polyQ fibril core decorated on two sides by exposed flexible flanking segments, akin to a two-sided hairbrush design^8,30^ (Figure 1B). Thus, there are two main types of surface features of HttEx1 fibrils that may be relevant. First, the water-facing surface of the block- or slab-like polyQ core itself. This core is multiple nm wide and assembled from multiple stacks of β-sheets, with at its surface the characteristic “grooved” surface typical of amyloid fibrils. It is this grooved surface that is the binding site for ThT and similar “amyloid” dyes. Alternatively, the flanking segments form a disordered and dynamic “fuzzy coat” on two sides of this rigid core. This fuzzy coat is formed by the proline-rich C-terminus of HttEx1 and the N-terminal Htt^NT^ segment. In prior work, especially the PRD has been implicated in mediating interactions between protofilaments in HttEx1 fibril bundles.^8,25,61^ An interesting finding in the HS-AFM results was that the secondary filaments remain attached to the seed fibrils, which differs from prior studies of Aβ42 aggregation where secondary protrusions were absent at the end of the reaction.^10^ The resulting fibril attachment is reminiscent of the side-by-side interfilament interactions noted in prior work.^8^ This suggests a role for flanking domain interactions in this interaction, which may be present from the initial stages of the secondary nucleation process. In this context, the experiments with the N-terminally truncated proteins are notable, as they argue against a dominant role for the Htt^NT^. This may be considered surprising, since especially Htt^NT^ is widely implicated in the early stages of HttEx1 aggregation and nucleation.^7,26,29^ Instead, this may point to a role for the proline rich C-terminal domain PRD. This may make sense, since this segment forms the bulk of the fibrillar fuzzy coat (being significantly larger than Htt^NT^ ^30^). However, it is also known to prevent or reduce (spontaneous) aggregation from HttEx1 monomers.^26,62,63^ Further HS-AFM experiments with HttEx1 with removed or modified PRD domains could help elucidate the role of this C-terminal domain in the aggregation process and in particular secondary nucleation.

Our results demonstrate the importance of both elongation of the fibril ends and secondary nucleation along the fibril surface in HttEx1 amyloid growth. The prevalence of secondary nucleation and subsequent branching suggests that the majority of volume is created through secondary nucleation. This finding is consistent with mechanistic analysis of aggregation kinetics, but also provides a unique molecular and real-time perspective of the process. Our mechanistic studies are enabled by work with purified proteins under controlled conditions, and in particular the application of advanced AFM. As in any “in vitro” study, it is conceivable that cellular conditions may have an impact that is not modeled in our approach, e.g., due to crowding or interactions with cellular membranes. Like other mechanistic studies, we relied on a cleavable fusion protein in our assays. In prior work, it has been shown that cleaved (MBP or GST) solubility tags do not significantly change the aggregation process.^64,65^ A key design feature in our protein construct is that cleavage by FXa does not leave extraneous N-terminal residues,^66^ or cause unintended cleavage events,^8^ both of which may affect aggregation kinetics.^65^ A C-terminal His-tag does remain in our construct, but this is part of the flexible C-terminal tail of the fibrils without clear effects on the fibrillar structure.^67^ Thus, prior studies do not suggest a major effect on the aggregation process, although more subtle effects may not be fully excluded in absence of further studies. At the same time, the presence of mica surfaces in our studies may have its own effects. It has for instance been shown that the hexagonal lattice of mica can induce directional growth of supramolecular structures,^68^ including amyloid-like assemblies.^69^ However, as the observed secondary nucleation events regularly seem to occur on top of fibrils, the presence of the mica surface is not expected to play a major role in this process. Future studies may help elucidate the extent to which these parameters play a significant role. Nonetheless, our results reinforce the idea that aggregation inhibitor studies should focus on prevention of secondary nucleation, as well as fibril elongation, in order to maximize therapeutic potential. While largely unexplored, to our knowledge, creating compounds that could coat or pacify those parts of the fibril surface involved in secondary nucleation could be very promising for interfering with protein aggregation and prion-like propagation processes. This would be the case for HttEx1, as studied here, but may be extendable to other disease-relevant amyloids that are prone to secondary nucleation.

## Conclusions

In summary, we have visualized in real time the elusive process of secondary nucleation of amyloids. Using High Speed AFM the dynamics of nucleation and elongation was captured at the single particle level. Focusing on Huntington’s disease, fibril formation of huntingtin Exon 1 HttEx1 was studied. The direct observations do not only reveal secondary nucleation, but also elongation and the formation of multifilament fibril clusters. While the N-terminal Htt^NT^ segment can enhance primary nucleation-aggregation, it does not seem to influence secondary nucleation. The visualized, complex aggregation process of HttEx1 and the role of secondary nucleation support the idea that developing therapeutic approaches via aggregation inhibitors should target secondary nucleation and fibril elongation. Finally, the presented approach can be widened for studies of other pathogenic protein misfolding events.

## Supplementary Material







## Notes

Example of growth of a fibril from a seed and a secondary nucleation event occurring on top of the newly grown part of the seed, the red arrow points at the site of secondary nucleation, snapshots of this movie are shown in Figure S11 (AVI)
Example of secondary nucleation and subsequent branching, the red arrows point at the sites of secondary nucleation, snapshots of this movie are shown in Figure 4D (AVI)
ThT-based Q32-HttEx1 aggregation kinetics analysis and modeling; TEM and kinetics analysis; transmission electron microscopy (TEM) data; High-Speed Atomic Force Microscopy (HS-AFM) data; HS-AFM snapshots of secondary nucleation and elongation; elongation profiles of newly formed secondary fibrils; HS-AFM snapshots of branching resulting in a broom-like structure (PDF)
