# Supplementary material for: Direct Observation of Secondary Nucleation in Huntingtin Amyloid Formation by High-Speed Atomic Force Microscopy
Source: J Am Chem Soc. 2025 Jun 12;147(25):21973–84. doi: 10.1021/jacs.5c05571 (PMC12203585; doi:10.1021/jacs.5c05571)
Supplement: Supplementary file 3 [file ja5c05571_si_003.pdf]

Supplementary Information

Direct observation of secondary nucleation in huntingtin amyloid formation by High-Speed Atomic Force Microscopy

C. van Ewijk[1], G. Jain[2], Y. K. Knelissen[1], S. Maity[1], P.C.A. van der Wel[2] and W.H. Roos[1,*]

[1] Molecular Biophysics, Zernike Instituut, Rijksuniversiteit Groningen, 9747 AG Groningen, The Netherlands

[2] Solid-State Nuclear Magnetic Resonance, Zernike Instituut, Rijksuniversiteit Groningen, 9747 AG Groningen, The Netherlands

* To whom correspondence should be addressed: w.h.roos@rug.nl

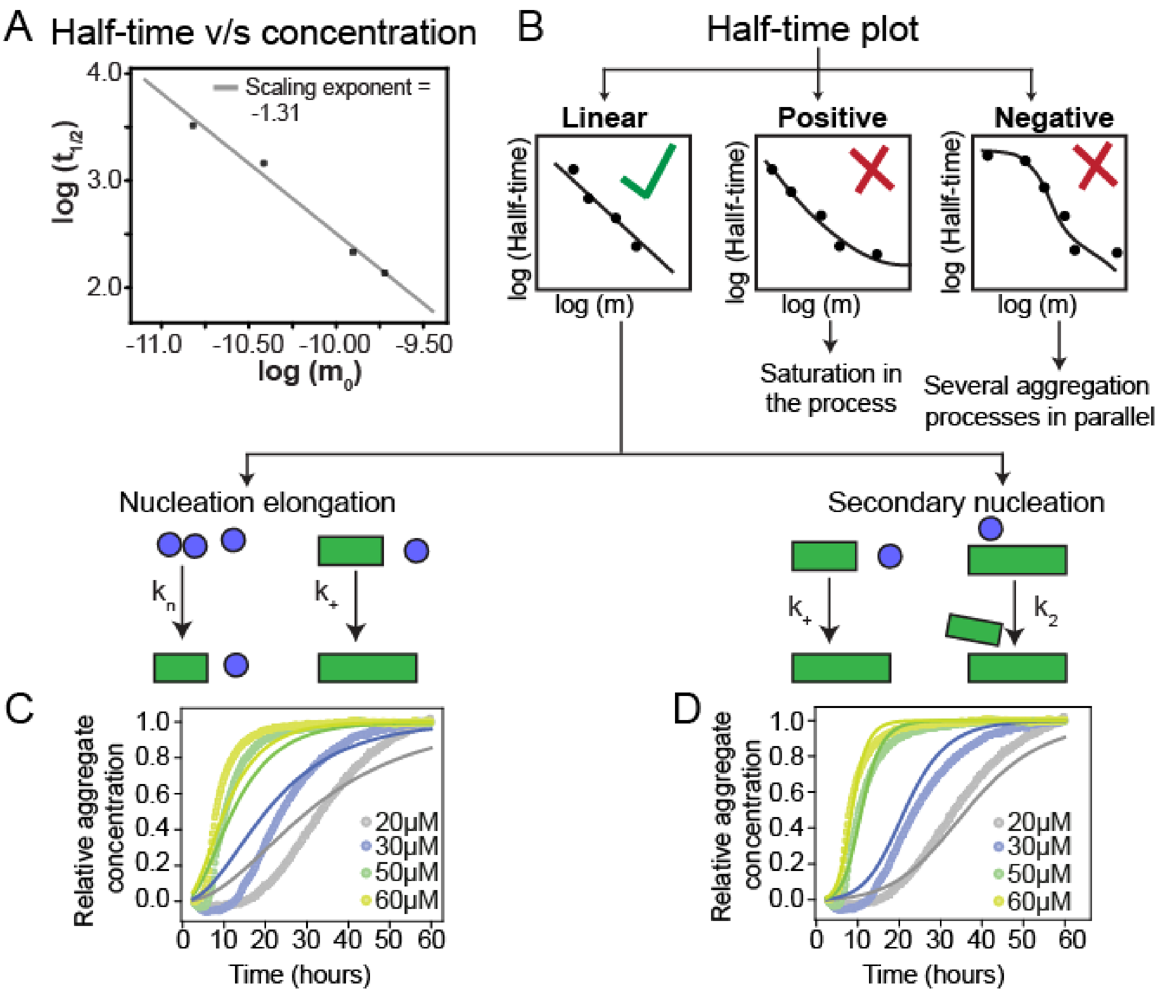

Figure S1. ThT-based Q32-HttEx1 aggregation kinetics analysis and modelling. **(**A). Log ($t_{1/2}$)- Log (m_0) plot of aggregation half-time versus concentration from the half-time plotter in the Amylofit program[1]. The linear curve shows that the monomer concentration does not change the dominant aggregation mechanism. The linear slope indicates a scaling component -1.31. (B) Schematic illustrations of possible deviations from a straight line, which would indicate that the scaling component depends on the concentration of the monomer and that the dominant aggregation mechanism changes with monomer concentration. The observed linear behavior means that one can apply a global fitting to the combined data curves. Below, we show schematics illustrating the modelled nucleation-elongation process (left) alongside models that also consider secondary nucleation (right). The constant k_n represents the rate at which monomeric protein (circles) spontaneously forms stable b-sheet nuclei (rectangle). These nuclei serve as a template for further aggregation. When a monomeric protein is added to the end of these nuclei, it is considered elongation (rate constant k_+). Secondary nucleation (rate k_2) refers to the formation of new nuclei facilitated by the presence of existing fibrils (but not simply elongation). This is thought to reflect the fibril surface catalyzing the formation of new nuclei. (C,D) Experimental ThT fluorescence data, along with fit curves (solid lines). The fitting values for each model are listed in Table S1. All ThT data were measured in triplicate, and the mean of the triplicates was plotted.

Table S1. Fitting parameters for nucleation elongation and secondary nucleation Amylofit models, from the data in Figure S1. For a detailed explanation of the parameters, see ref. Meisl et al (2016)[1].

	**Nucleation Elongation**		**Secondary Nucleation**
k_+k_n	$2.37e^{+6}$ conc^{-nc} time^{-2}	k_+k_n	$4.9e^{+5}$ conc^{-nc} time^{-2}
n_c	2	n_c	2
Mean Residual Error	0.0113	k_+k_2	$1.09e^{+12}$ conc^{-nc} time^{-2}
		n_2	2
		Mean Residual Error	0.00372

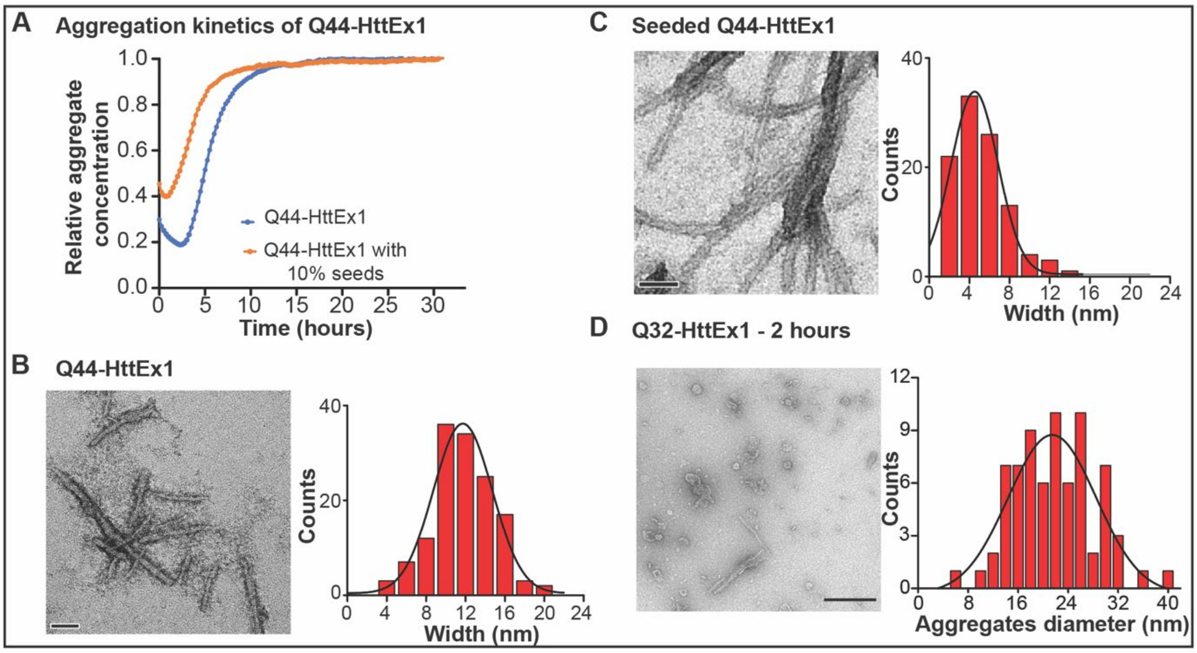

Figure S2. TEM and kinetics analysis of Q44-HttEx1 aggregation and TEM data on non-fibrillar Q32-HttEx1 aggregates. (A) Aggregation kinetics of 67.5 µM of Q44-HttEx1 protein with and without 10% Q44-HttEx1 fibril seeds. The ThT data were measured in triplicate and the mean of the triplicates was plotted. (B) Negative stain TEM images of 45 µM of Q44-HttEx1 fibrils, and (C) 45 µM of Q44-HttEx1 seeded with 10% Q44-HttEx1 preformed fibrils, aggregated at room temperature for 96 hours. The width of fibrils in (B) is 12 ± 3 nm, ns=139, and (C) is 5 ± 2 nm, ns=102. The scale bars in B-C are 50 nm. (D) TEM image and diameter analysis of globular non-fibrillar aggregates at 2h time interval for Q32-HttEx1 aggregation. The scale bar in D is 0.2 µm.

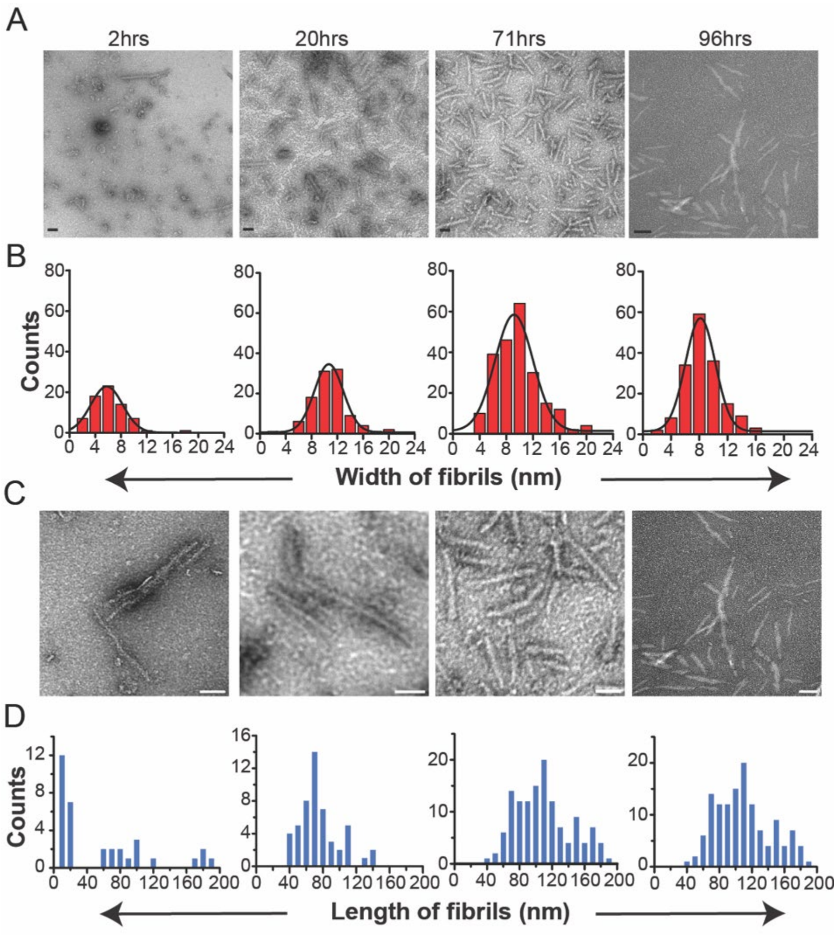

Figure S3. Transmission electron microscopy (TEM) data of Q32-HttEx1 aggregation. (A,C) Negatively stained TEM micrographs of 51 μM of Q32-HttEx1 aggregates at respective time intervals of 2, 20, 71, and 96 hours. The scale bar is 50 nm. B) Fibril width histograms are based on an analysis of the TEM data. D) Fibril length histograms based on an analysis of the TEM data.

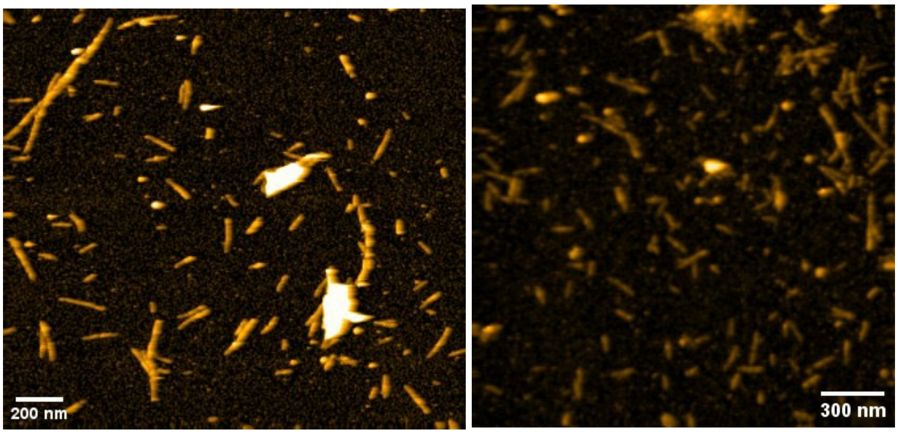

Figure S4. HS-AFM images of Q44-HttEx1 fibrils. Two representative overview images are depicted, showing the distribution of sizes of the fibrils. More zoomed-in images are shown in figure 2A and 2B of the main manuscript.

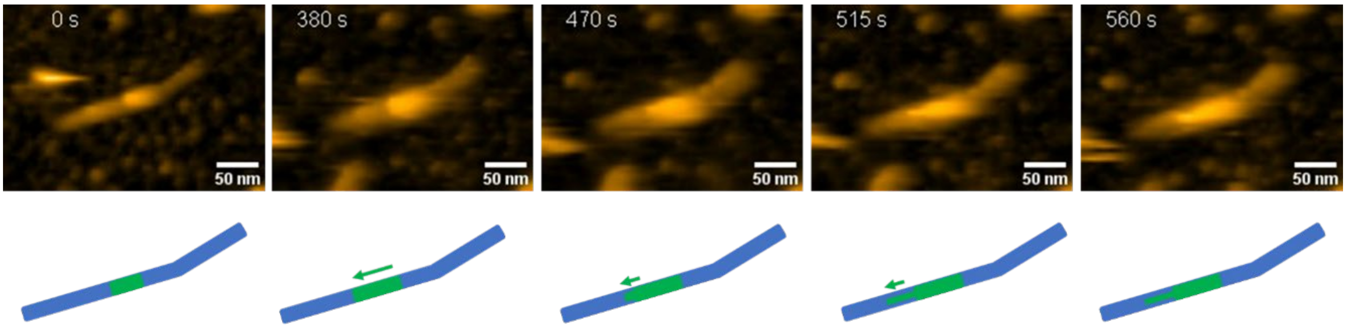

Figure S5. Secondary fibril growth on the surface of a Q44-HttEx1 amyloid fibril as shown in panel 3E, with accompanying schematic below. The green segment indicates the secondary fibril and the green arrow indicates the growth direction.

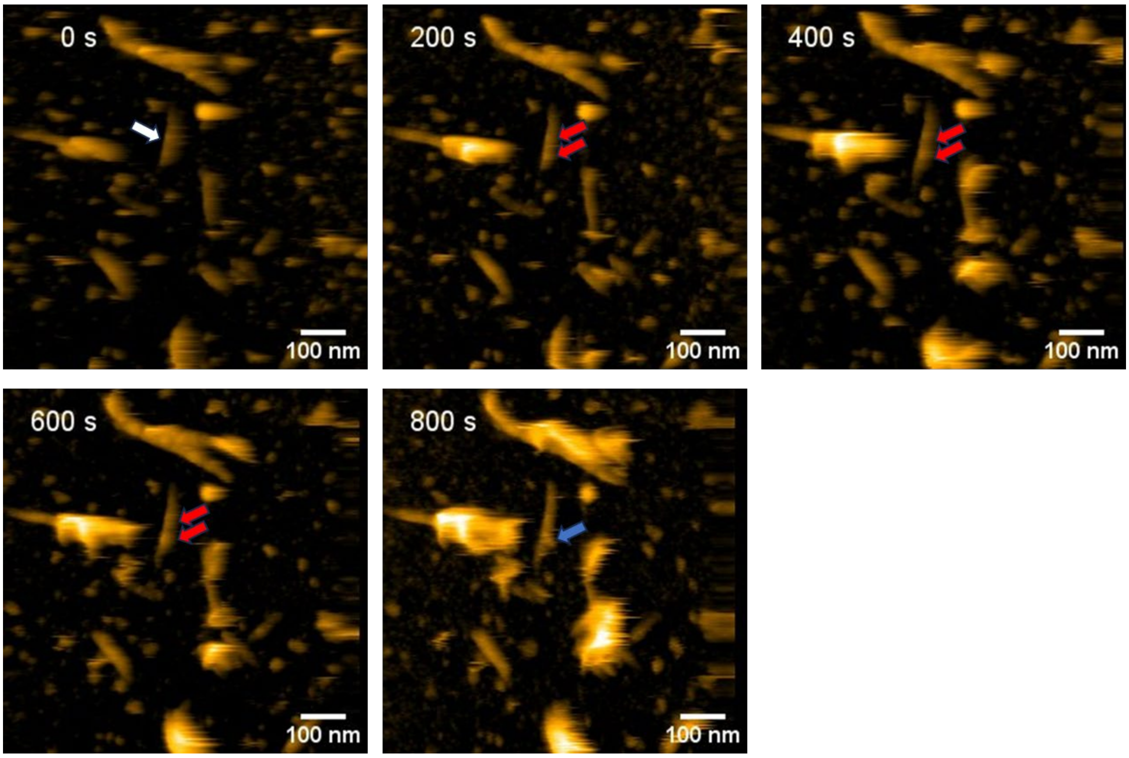

Figure S6. Imaging of this sample started at 0s and while elongation, secondary nucleation and branching can be observed throughout the whole sample, we focused on one fibril in the following description for clarity. 0s: the white arrow denotes a seed from which a fibril elongates. In the subsequent images it can be seen that this fibril gets longer. 200s: the red arrows show two secondary nucleation events on the fibril. 400s & 600s the red arrows denote the same two secondary nucleation events as in the 200s image. It can be observed that the secondary nucleated fibrils are elongating. 800s: The blue arrow denotes the lower secondary nucleated fibril, which is branching of the primary fibril surface.

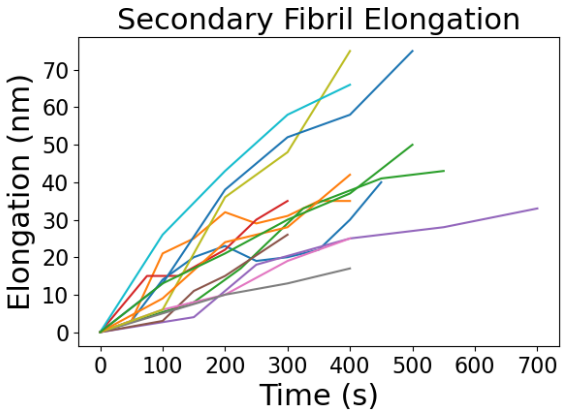

Figure S7. Elongation profiles of newly formed secondary fibrils. The graphs show for each secondary fibril the length as a function of time (n=13). While on roughly 90% of the fibrils we observe secondary nucleation, due to experimental constraints it was not possible to follow all of them over a long enough time frame. In this graph 13 secondary fibrils that were followed for an extended period of time are analysed. The analysed secondary fibrils are the same fibrils as analysed for figure 3D in the main text.

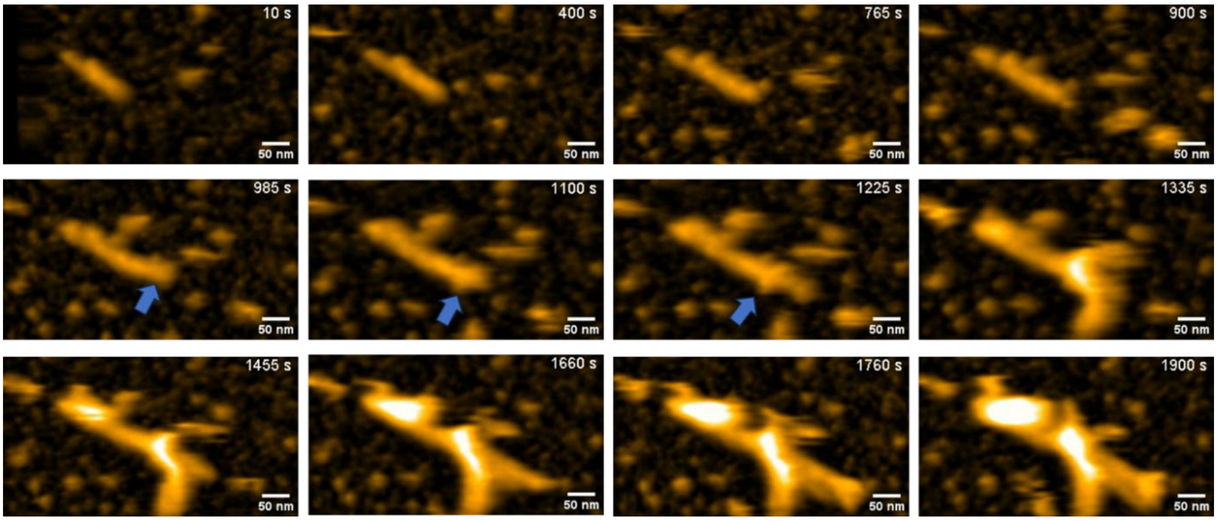

Figure S8. Snapshots of a growing Q44-HttEx1 amyloid fibril. The data is the same as shown in panel 3A, but more snapshots are shown. It reveals a secondary nucleation event that occurs on a freshly elongated fibril, indicated by the blue arrows. The secondary nucleated fibril grows and branches.

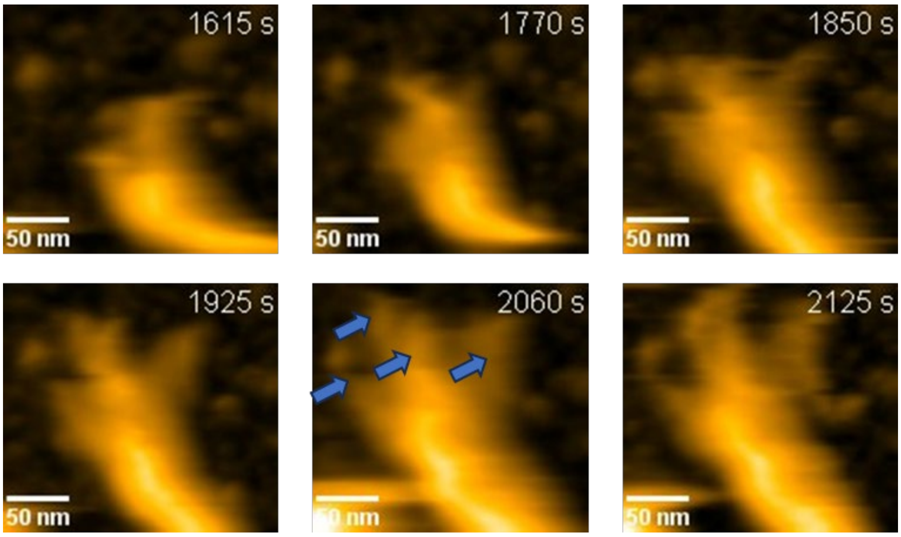

Figure S9. Snapshots of branching resulting in a broom-like structure. The broom-like structure is especially apparent in the bottom three images where at the top left of the images one can observe three to four fibrils (blue arrows in 2060 s image).

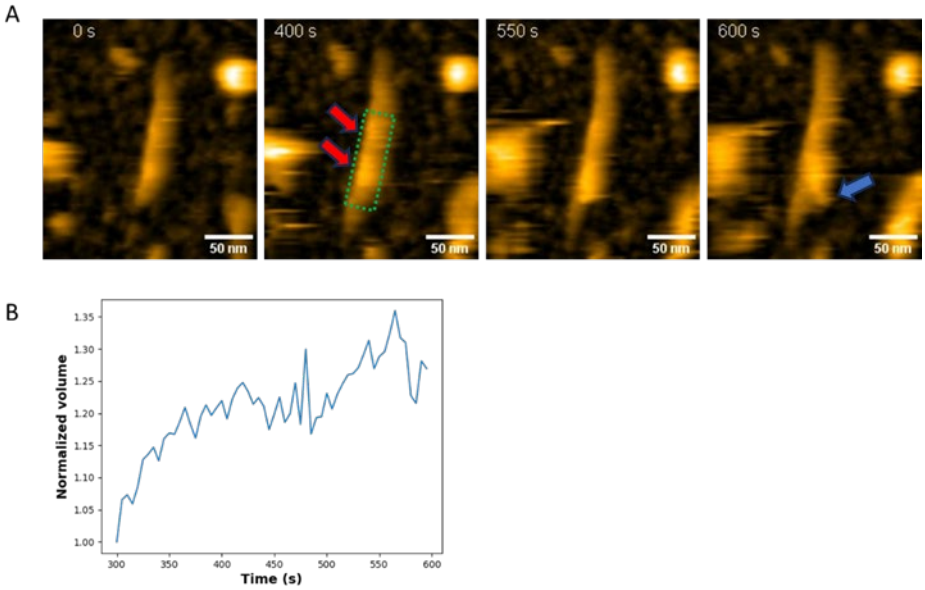

Figure S10. A) Secondary nucleation of Q44-HttEx1. Two nucleation sites are present on the parent fibril and grow in size and length over time. One of the secondary fibrils branches off from the parent structure at 600s. B) Normalized volume increase over the selected area in A that is highlighted by the green dashed box.

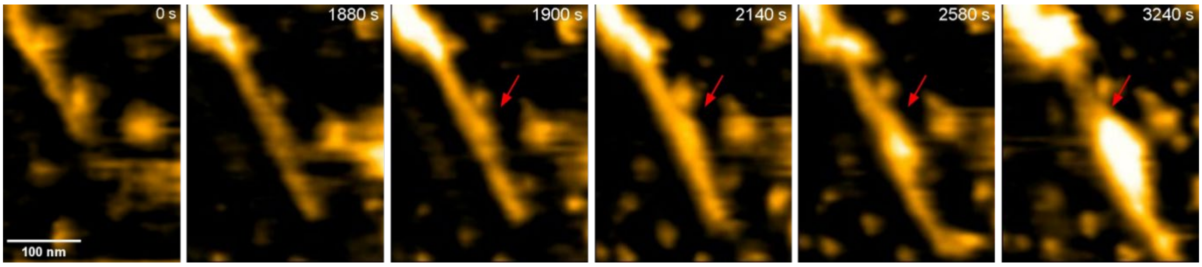

Figure S11. Snapshots of movie S1 where growth of a Δ15-Q44-HttEx1 amyloid fibril from a seed is seen and where a secondary nucleation event occurs on top of the newly grown part of the seed. The red arrow points at the site of secondary nucleation.

SI movies

Movie S1: Example of growth of a fibril from a seed and a secondary nucleation event occurring on top of the newly grown part of the seed. The red arrow points at the site of secondary nucleation. Snapshots of this movie are shown in Fig. S11

Movie S2: Example of secondary nucleation and subsequent branching. The red arrows point at the sites of secondary nucleation. Snapshots of this movie are shown in Fig. 4D

References cited in the Supporting Information

(1) Meisl, G.; Kirkegaard, J. B.; Arosio, P.; Michaels, T. C. T.; Vendruscolo, M.; Dobson, C. M.; Linse, S.; Knowles, T. P. J. Molecular Mechanisms of Protein Aggregation from Global Fitting of Kinetic Models. *Nat Protoc* 2016, *11* (2), 252–272. https://doi.org/10.1038/nprot.2016.010.
